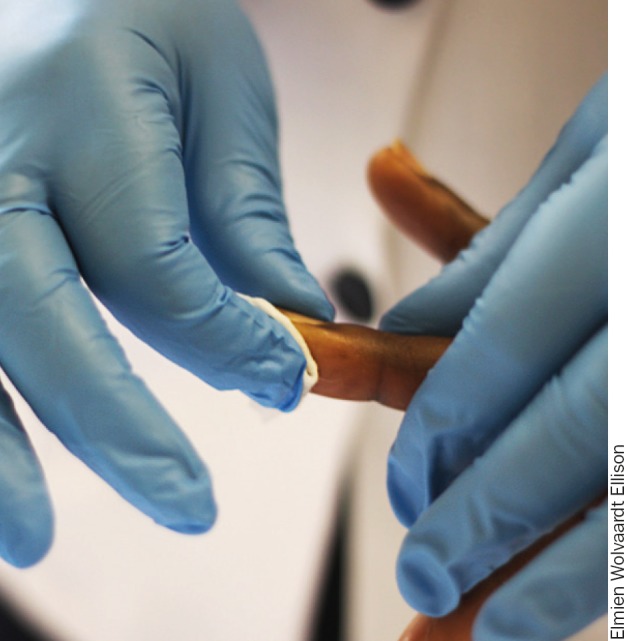# How to measure blood glucose

**Published:** 2014

**Authors:** Dianne Pickering, Janet Marsden

**Affiliations:** Nurse Advisor (retired): *Community Eye Health Journal*dianne_logan@hotmail.com; Nurse Advisor: *Community Eye Health Journal*, London, UK. Email: J.Marsden@mmu.ac.uk

**Figure:**
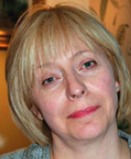
Dianne Pickering

**Figure:**
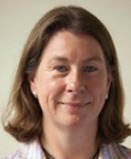
Janet Marsden

## Background

The level of glucose in the blood can be measured by applying a drop of blood to a chemically treated, disposable ‘test-strip’, which is then inserted into an electronic blood glucose meter. The reaction between the test strip and the blood is detected by the meter and displayed in units of mg/dL or mmol/L. There are a number of different types of meters available, and all are slightly different. Take care when applying the general principles described in this article to the specific glucose meter you are using.

### Why measure blood glucose?

- It can be used as a screening tool for diabetes mellitus (diabetes).
- It is an important tool in the assessment of the unwell patient, especially in the young or old.
- Potentially life-threatening extremes of blood glucose can be detected to enable the patient, carer or health worker to respond to high (hyperglycaemia) and low (hypoglycaemia) blood glucose by adjusting the diet or using insulin.

### When to measure blood glucose

- Blood glucose should be measured whenever your patient with diabetes is feeling unwell in any way.
- In the diabetic patient, it should be measured before surgery to ensure that the patient is not going be become unwell during surgery and/or after general anaesthetic. Measure regularly until the patient is eating and drinking normally and blood glucose is stable.
- In newly diagnosed diabetes patients, more frequent measurements are needed, until blood glucose is stable.

**Top tips**

**Patient safety and comfort**

- Be aware of what ‘normal’ blood glucose levels are. Find out what is ‘normal’ for individual patients by asking them and/or checking their notes or file.
- Take universal precautions as blood is being handled.
- Use aseptic techniques as the skin is being punctured. While it would be unusual for infection to occur, patients with diabetes tend to heal less well and may not deal as well with infection.
- Invite the patient to do the procedure if they self-test regularly (provided they are familiar with the particular meter); they may well be better at it than the health worker. Take the opportunity to check the patient's technique.
- Do take notice if the patient gives you ideas about where best to take blood from!

**If you can't get blood from the finger prick**

- Ask the patient to hang the hand down below the waist for a minute or two.
- Ask the patient to place the hands in or under warm water and rub them together.
- Grasp the area to be pricked and squeeze gently for 3 seconds.
- Place the finger on a table or other firm surface to avoid moving while pricking.
- If the lancing device has a dial-a-depth facility, increase the setting by 1 level.

**NOTE:** Blood glucose **monitoring** is done to measure the concentration of glucose in the blood (glycaemia) **over time**, and is important in the care of patients with diabetes mellitus. Information about individual patterns of blood glucose changes, gathered through blood glucose monitoring, can be used to plan meals, activities, and at what time of day to take insulin. The better the patient's blood glucose control, the less likely it is that the diabetes will cause damage in the body and lead to complications such as loss of vision (due to diabetic retinopathy) and amputation.

## You will need

- Blood glucose monitor
- Test strips (check that they are in date and have not been exposed to the air)
- Alcohol swab
- Single-use safety lancets or lancing device
- Gloves
- Cotton wool/gauze
- Sharps box
- Control solution for calibration

## Method

Apply these general principles when using the different types of electronic blood glucose meters available.

- Ask the patient to sit down and explain what you are going to do.
- Wash your hands and put on gloves.
- Choose the site for the blood sample: usually the side of a finger, but the arm or thigh may be used (change the site used if frequent measurements are needed).
  **Figure 1:**
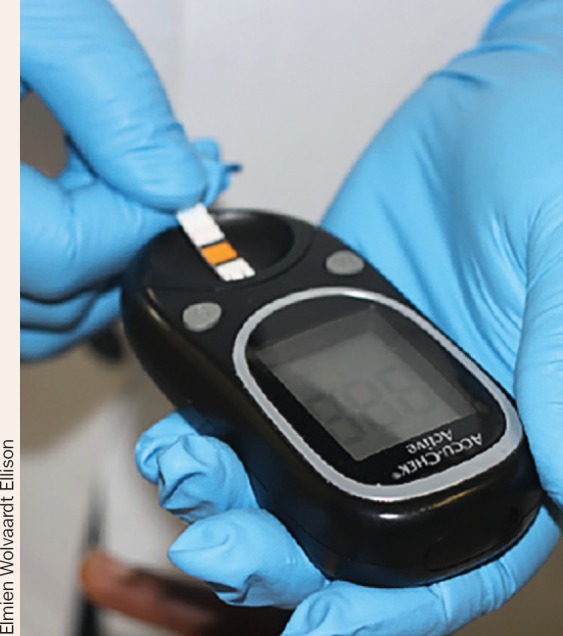

**Figure 2:**
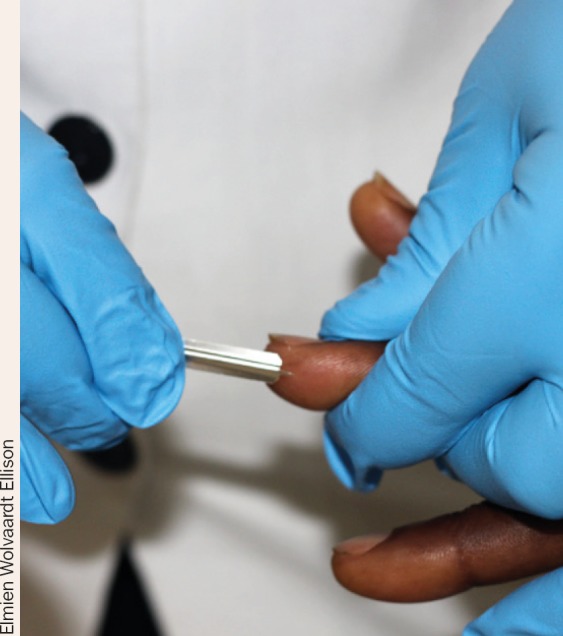

**Figure 3:**
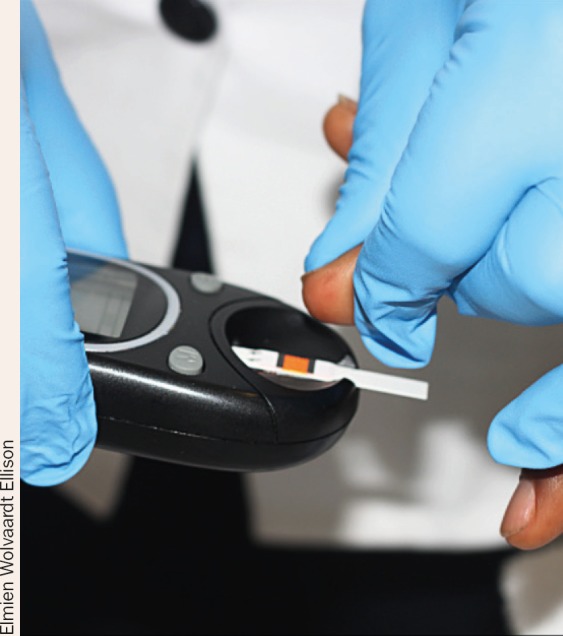
- Use an alcohol swab to clean the site and let the alcohol dry.
- Insert the test strip into the monitor, following the instructions (Figure 1).
- Use a single-use lancet or a lancing device to draw blood (Figure 2) and dispose of it in a sharps container.
- Apply the blood to the testing strip (Figure 3) in the correct way: some strips need the blood drop to be over the whole of the test pad and some suck up the blood directly from the site of the bleeding.
- Place the alcohol swab (note: it will sting) ora piece of gauze over the site (Figure 4) and hold it there, or let the patient hold it there until the bleeding stops. Monitor for excess bleeding.
- Read and record the result, reporting and/or responding to abnormal readings.
- Tell the patient what the result is, explain it and discuss options.
- Dispose of all used equipment safely, in line with hospital or health care policies.

**Calibrating the blood glucose monitor**

- Calibrate the monitor and each new pack of test strips together.
- Calibrate the monitor each week.
- Place the control solution on a test strip and check that the value shown on the monitor matches the value on the bottle (or the pack of strips it accompanies). Record the calibration readings.
- If one is provided, use the check strip to make sure that the meter is working.

**Figure 4:**